# A strategic model of a host–microbe–microbe system reveals the importance of a joint host–microbe immune response to combat stress-induced gut dysbiosis

**DOI:** 10.3389/fmicb.2022.912806

**Published:** 2022-08-04

**Authors:** István Scheuring, Jacob A. Rasmussen, Davide Bozzi, Morten T. Limborg

**Affiliations:** ^1^Centre for Ecological Research, Institute of Evolution, Budapest, Hungary; ^2^MTA-ELTE, Research Group of Theoretical Biology and Evolutionary Ecology, Eötvõs University, Budapest, Hungary; ^3^Center for Evolutionary Hologenomics, GLOBE Institute, University of Copenhagen, Copenhagen, Denmark; ^4^Department of Computational Biology, University of Lausanne, Lausanne, Switzerland; ^5^Swiss Institute of Bioinformatics, Lausanne, Switzerland

**Keywords:** bistability, mutualism, stress, pathogens, salmonids, microbiome, *Mycoplasma* sp., *Aliivibrio* sp.

## Abstract

Microbiomes provide key ecological functions to their host; however, most host-associated microbiomes are too complicated to allow a model of essential host–microbe–microbe interactions. The intestinal microbiota of salmonids may offer a solution since few dominating species often characterize it. Healthy fish coexist with a mutualistic *Mycoplasma* sp. species, while stress allows the spread of pathogenic strains, such as *Aliivibrio* sp. Even after a skin infection, the *Mycoplasma* does not recover; *Aliivibrio* sp. often remains the dominant species, or *Mycoplasma*–*Aliivibrio* coexistence was occasionally observed. We devised a model involving interactions among the host immune system, *Mycoplasma* sp. plus a toxin-producing pathogen. Our model embraces a complete microbiota community and is in harmony with experimental results that host–*Mycoplasma* mutualism prevents the spread of pathogens. Contrary, stress suppresses the host immune system allowing dominance of pathogens, and *Mycoplasma* does not recover after stress disappears.

## Introduction

Almost every eukaryotic organism hosts an associated core microbial community providing key biological functions to the host (McFall-Ngai et al., 2013; Bosch and Miller, 2016; Müller et al., 2016). This has led influential thinkers to coin the term holobiont as describing the sum of a host and its commensal microbes (Margulis, 1990; Baedke et al., 2020). These host–microbiota systems range in complexity from one-to-one symbiotic associations between a host and a single microorganism, such as the bioluminescent *Aliivibrio* bacteria in light organs of bob-tail squids (Nyholm and McFall-Ngai, 2004), to intricate arrangements between a host and a dynamic community of microorganisms like vertebrates and their gut microbiota (Ley et al., 2008), or plants and their root microbiota (Sasse et al., 2018). The renewed realization that microbes play essential roles for the hosts has catalyzed an increased focus on the study of host–bacteria and bacteria–bacteria dynamics within a holobiont (Zilber-Rosenberg and Rosenberg, 2008; Bordenstein and Theis, 2015; Theis et al., 2016). In extension, the generation of knowledge potentially allowing active manipulation of holobionts has become a global strategic priority across life sciences (Małyska et al., 2019), including food production (Limborg et al., 2018).

One approach to better understand microbiome dynamics is ecological models that include a realistic parameter space for characterizing host–microbe interactions in the holobiont. Microbiomes of animal hosts are generally very complex (Gralka et al., 2020; Alberdi et al., 2021). So far, theoretical studies have achieved limited success in explaining empirical data of these complex systems. Even verification of more simplified feedback and dynamical models describing host–microbe interactions remains scarce (Abbott et al., 2021; Remien et al., 2021). The challenge becomes even more significant if we model how pathogenic microbes interact with the host and the host's commensal and mutualistic contingent of the host–microbiome dynamics (Coyte et al., 2015; Rúa and Umbanhowar, 2015; Jiang et al., 2020). Indeed, to adequately describe realistic host–microbiome dynamics, models must consider at least two key factors that have been ignored in attempts to model realistic holobiont systems reflecting empirical data. First, the host immune system needs to be included as it is known to control microbiome composition (Earley et al., 2018; Zheng et al., 2020). Second, microbial metabolites can act as toxins, common goods, or resources, further shaping the qualitative dynamics of the system (Scheuring and Yu, 2012; Rybicki et al., 2018; Kokou et al., 2019; Gralka et al., 2020). We address this challenge by studying a holobiont system containing relatively few microbial members while covering the complete microbiome community.

Recent investigations have revealed a general trend of low diversity among intestinal microbiota in teleosts compared to warm-blooded animals, including numerous studies from commercially important species such as Atlantic salmon (*Salmo salar*) and rainbow trout (*Oncorhynchus mykiss*) (Huang et al., 2020). Adult salmon are piscivorous and characterized by physiological adaptations necessary to cope with a strictly carnivorous diet. These adaptations may extend to an adaptive composition of its associated gut microbiota. Furthermore, several studies have revealed that the intestinal microbiota of salmonids is characterized by strikingly low diversity, with as little as one or two species dominating the microbial biomass (Holben et al., 2002; Llewellyn et al., 2016; Bozzi et al., 2021; Wang et al., 2021). Together, these observations suggest that salmon and related species are well-suited holobiont systems to study concrete biological interactions between a eukaryotic host and its commensal microbiota (Limborg et al., 2018; Nyholm et al., 2020; Alberdi et al., 2021).

*Mycoplasma* sp. has recently emerged as a core and often dominating member of the gut microbiome in some salmonid species. This novel *Mycoplasma* species have been reported at high-relative abundances in the gut of different salmonids species in numerous independent studies over the past 20 years (Holben et al., 2002; Zarkasi et al., 2014; Lowrey et al., 2015; Llewellyn et al., 2016; Dehler et al., 2017; Lyons et al., 2017; Brown et al., 2019; Rimoldi et al., 2019; Bozzi et al., 2021; Rasmussen et al., 2022a,b). *Mycoplasma* sp. abundance has been associated with enhanced health conditions (Bozzi et al., 2021), disese resilience (Rasmussen et al., 2022b), and improved growth performances (Rimoldi et al., 2019; Bozzi et al., 2021) of the salmonid host. More detailed studies using genome-resolved metagenomics further point toward a putative mutualistic relationship between *Mycoplasma* sp. and its salmonid hosts (Cheaib et al., 2021a; Rasmussen et al., 2021). For example, *Mycoplasma* sp. can provide the host with a suite of beneficial functions, such as arginine biosynthesis, ammonia detoxification, and degradation of long-chain polymers, which could improve the nutritional value of both chitin-rich diet and strict carnivory during the juvenile and adult life stages of salmon (Rasmussen et al., 2021).

Interestingly, the proposed beneficial role of *Mycoplasma* sp. is further supported by numerous observations where slower-growing or disease-susceptible salmonid cohorts have a reduced abundance of *Mycoplasma* sp. in concomitance with the increase of pathogenic/opportunistic strains (Table 1). These observations, together with the resolved *Mycoplasma* phylogeny (Rasmussen et al., 2021), suggest a mutualistic relationship, thus providing an excellent system to further model and understand adaptively important host–microbe and microbe–microbe interactions. Here, we build upon a previous case study to develop a simple mathematical model describing the dynamics of an observed change in *Mycoplasma* sp. abundance in a sick cohort of Atlantic salmon.

**Table 1:** A non-exhaustive list of relevant studies showing similar positive correlations with *Mycoplasma* sp. abundance and fish health.

**Host species**	**Type of stress**	**Microbiota pattern (before and after stress)**	**References**
Atlantic salmon	Tenacibaculosis outbreak	*Mycoplasma* dominates healthy control fish while its abundance is highly reduced in diseased fish.	Bozzi et al., 2021
Rainbow trout	*Yersinia ruckeri* challenge	*Mycoplasma* dominates healthy control fish while its abundance is highly reduced in diseased fish.	Rasmussen et al., 2022b
Atlantic salmon	Tenacibaculosis outbreak	The intestinal microbiota initially dominated by *Mycoplasma* experienced an increase in *Aliivibrio* and *Alcaligenes* abundance in the intestine of fish with ulcerative disorder.	Karlsen et al., 2017
Rainbow trout	Comparison of the microbiome of a selectively bred line resistant to *Flavobacterium psychrophilum* infection with the susceptible line.	*Mycoplasma* sp. was the dominant taxon in the midgut of both groups, although, in the susceptible line, it was present at a decreased abundance, together with an increased abundance of the potential opportunistic pathogen *Brevinema andersonii*.	Brown et al., 2019
Rainbow trout	The Effects of a dietary insect meal	The relative abundance of Aeromonadaceae (a family that includes pathogenic species) decreased in fish fed with higher percentages of insect meal. Concurrently mycoplasmataceae amount increased in these samples.	Rimoldi et al., 2019
Chinook salmon (*Oncorhynchus tshawytscha*)	No stress reported	The mid-intestinal microbiota of the majority of the 30 sampled fish was dominated by the family Vibrionaceae, except two of the individuals which had a microbiota dominated by Mycoplasma.	Ciric et al., 2019

**Figure 1:**
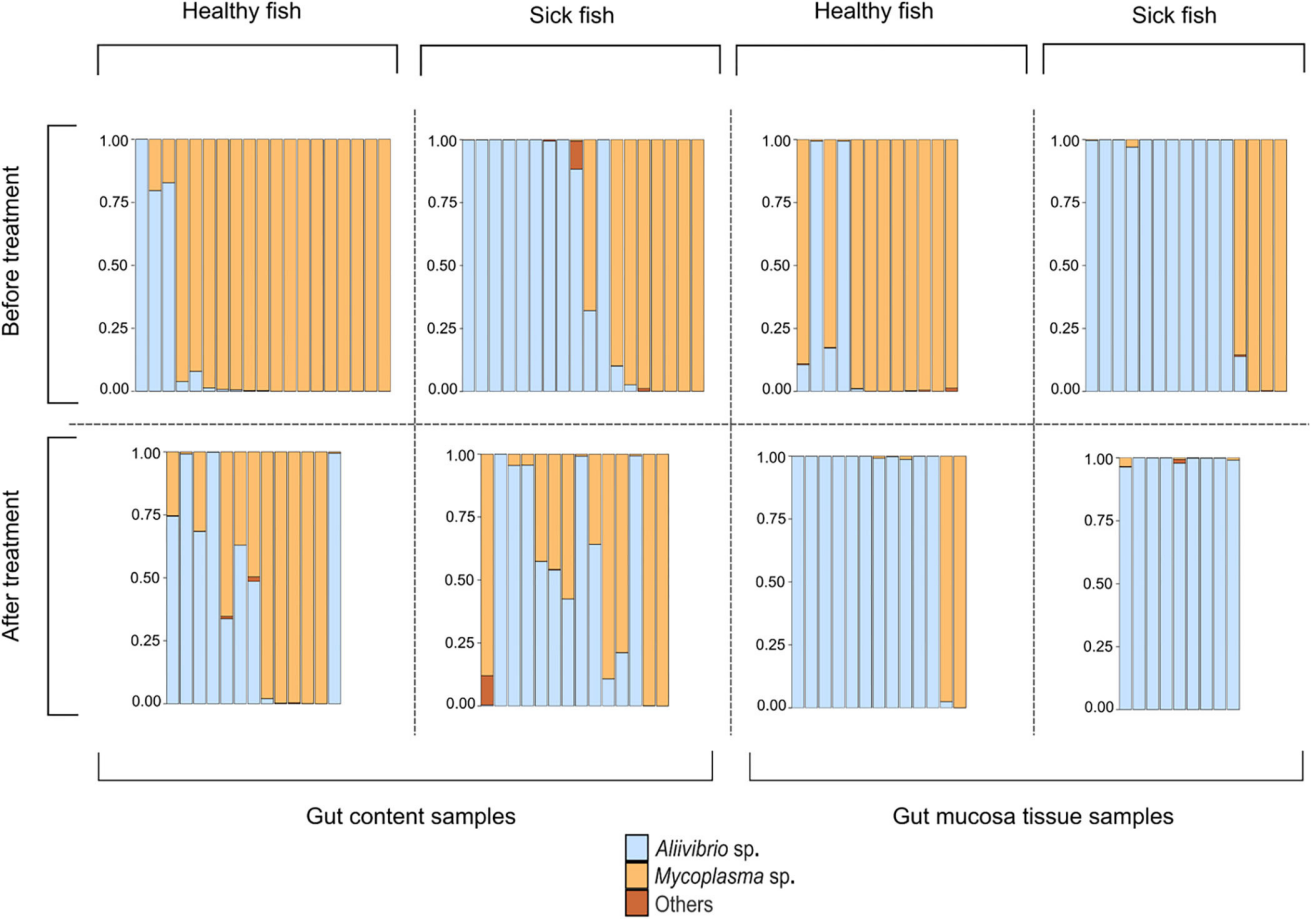
Intestinal microbiota composition for the two dominant bacterial species *Mycoplasma* sp. and *Aliivibrio* sp. for eight distinct cohorts of Atlantic salmon during a disease outbreak. Each bar represents one fish. The eight cohorts represent three relevant variables pertaining to the sampled tissue (gut content vs. gut mucosa), the health status during a Tenacibaculosis outbreak (Healthy vs. Sick), and whether fish were sampled before or after treatment against the Tenacibaculosis causing pathogen *Tenacibaculum dicentrarchi* (before vs. after treatment). The figure has been reformatted based on data from Bozzi et al. (2021).

The study of Bozzi et al. (2021) provides a valuable dataset to describe a model involving interactions among the host immune system, *Mycoplasma* sp. plus a toxin-producing pathogenic competitor as the two dominant gut microbes. Bozzi et al. (2021) assessed changes in the composition of the Atlantic salmon distal gut microbiota in the context of a bacterial skin infection caused by the pathogen *Tenacibaculum dicentrarchi*. The infected fish developed an ulcerative skin disease, which would eventually lead to the death of the fish. The researchers collected samples from the distal gut content and the distal gut mucosa tissue of both healthy and diseased salmon. The stressful event was resolved by a water disinfection treatment aimed at killing the skin pathogen. The sampling procedure was then repeated after treatment. The microbiome composition was investigated with 16S rRNA amplicon sequencing and described the relative abundance of dominating microbial species (Figure 1). Before treatment, most healthy fish had a *Mycoplasma*-dominated gut microbiome. The infection, even if it affects the outer skin of the host, allows the spread of an opportunistic and potentially pathogenic *Aliivibrio* strain, leading to its dominance in the gut of the sick fish. These observations were consistent with the gut content and mucosa tissue samples. After treatment of the infection, the *Mycoplasma*-dominated microbiomes of healthy fish do not recover, and *Aliivibrio* sp. remains the dominant species in most of the samples of the gut mucosa tissue. Instead, in the gut content, we observe the presence of some samples showing patterns of *Mycoplasma*–*Aliivibrio* coexistence (Figure 1).

In this study, we consider the study of Bozzi et al. (2021) as a case to model and understand the dynamics of a concrete host–microbe–microbe model exemplified by the *Mycoplasma*-dominated salmonid microbiomes in the context of a stressful event and the emergence of an opportunistic/pathogenic bacteria.

Based on the experimental observations described above, we define the following assumptions about the case study for building our model:

1. Salmon and *Mycoplasma* form a mutualistic relationship (Rasmussen et al., 2021), so we assume that the immune system of the host increases the carrying capacity of the *Mycoplasma* in the distal gut, and vice versa the presence of *Mycoplasma* activates the immune system either directly or indirectly by keeping the host in a healthier state (Cerf-Bensussan and Gaboriau-Routhiau, 2010; Pérez et al., 2010; Koch and Schmid-Hempel, 2011; Earley et al., 2018; Xiong et al., 2019).
2. *Aliivibrio* is a putative toxin-producing pathogen of salmonids. The assumption is based on the fact that the known *Aliivibrio* sp. generally infects its host with the help of a toxin by suppressing the immune system (Shinoda, 1999; Karlsen et al., 2014; Pérez-Reytor et al., 2018). We build our assumption on these studies to allow the pathogenic species to exert a negative impact on mutualistic bacteria in the model.
3. *Mycoplasma* colonizes the intestine of salmonids in the juvenile phase (Cheaib et al., 2021a; Rasmussen et al., 2022b) before the *Aliivibrio* can infect it. Alternatively, it can be the case that *Aliivibrio* infection in the juvenile phase is highly lethal for the host, which does not modify our argument below.
4. *Mycoplasma* and *Aliivibrio* compete in the distal intestine; that is, space and nutrients are common limiting factors of these two species. Additionally, *Aliivibrio* can also be toxic for *Mycoplasma*, which is considered in the model.
5. Infection or other stress factors elicit an acute immune response that will remove resources from other fish metabolic processes, including transcription of host genes usually involved in maintaining gut homeostasis in the host fish (Tort, 2011; Nardocci et al., 2014; Cámara-Ruiz et al., 2021).

## Materials and methods

**Figure 2:**
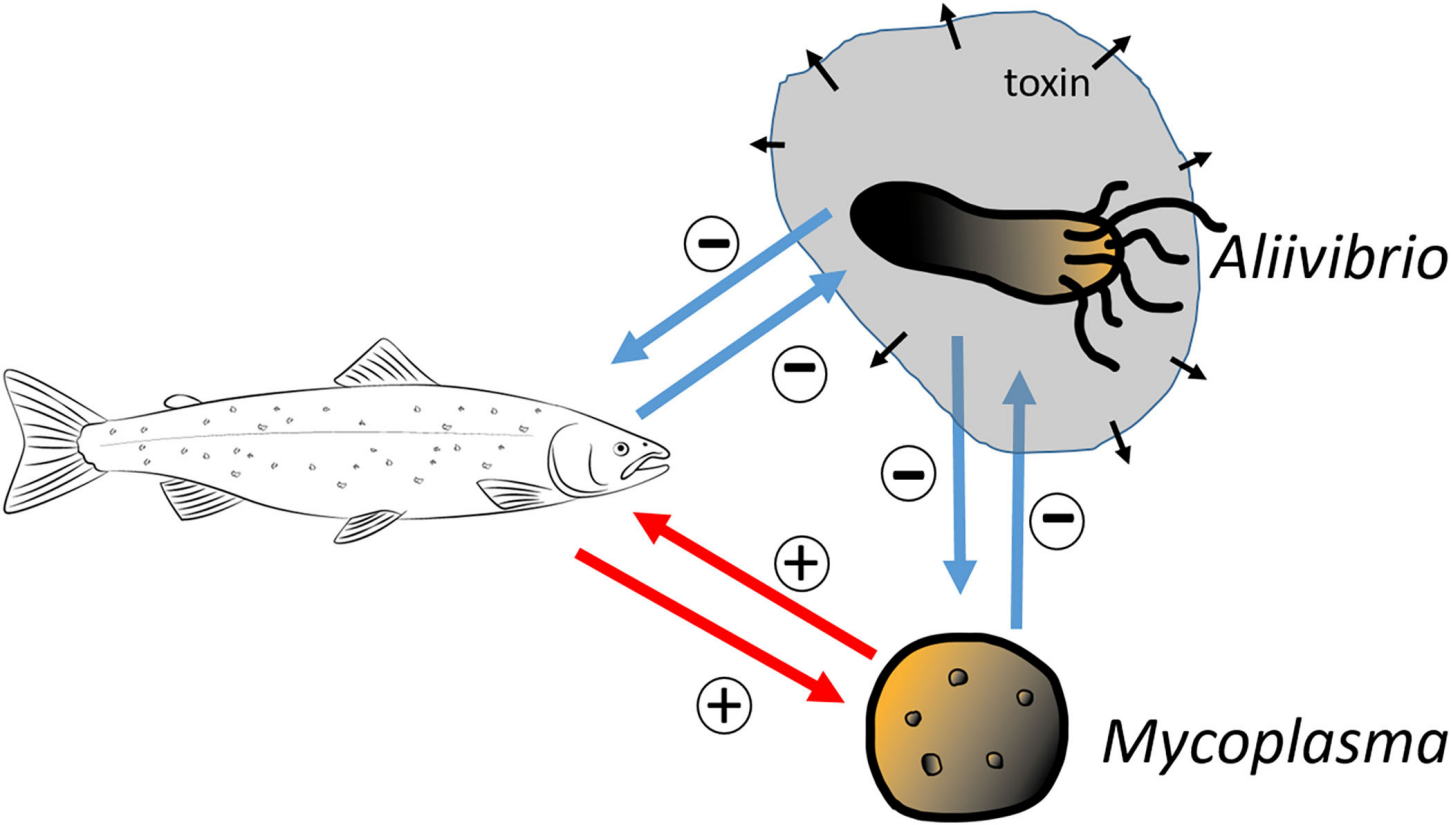
A conceptual schematic of the model. The salmon and *Mycoplasma* engaged in mutualistic interaction with each other (+ signs at the red arrows). Conversely, the toxin-producing *Aliivibrio* harms the salmon, which in turn defends itself *via* its immune response (– signs at the blue arrows). The Mycoplasma and Aliivibrio species of the model compete for the same niches and resources so that the expansion of one species is at the expense of the other species (– signs at the blue arrows).

We consider a simple dynamical model to describe the dynamics of the host immune system, the mutualistic microbe, and the invading toxic producing bacterium. As we argued above, in the case of salmonid hosts, the resident microbiome is typically dominated by *Mycoplasma*. Still, after some stress, the microbiome is frequently replaced by an opportunistic pathogenic *Aliivibrio* species. We apply the common Lotka–Volterra competition model to describe the *Mycoplasma–Aliivibrio* competition in their common habitat. We define a model where the mutualistic *Mycoplasma* facilitates the immune system of the host; in return, the host's immune system selectively helps to maintain a higher density of *Mycoplasma* in the gut. Furthermore, we use a simple model for the pathogen-immune sub-system, where the *Aliivibrio* pathogen produces toxins that inhibit immune response while immune effectors try to eliminate pathogens (Rybicki et al., 2018). Figure 2 depicts the interactions between the microbes and host, and the corresponding dynamical system is the following:


(1a) $$\begin{array}{r} \frac{dA}{dt}\;=\;r_{A}\left(1-\frac{A}{K_{a}}-a_{MA}M\right)A-kIA \end{array}$$

(1b) $$\begin{array}{r} \frac{dM}{dt}\;=\;r_{M}\left(1-\frac{M}{K_{M}\left(I\right)}-a_{AM}A\right)M \end{array}$$

(1c) $$\begin{array}{r} \frac{dT}{dt}\;=\;sA-mT \end{array}$$

(1d) $$\begin{array}{r} \frac{dI}{dt}\;=\;r\left(I_{0}\left(M\right)-I\right)-eIT, \end{array}$$

where *A* and *M* are the concentration of *Aliivibrio* and *Mycoplasma* species in the gut of the host, *T* and *I* are the concentration of toxin and immune effectors, *r* and s are the growth rates of microbes and the effector cells, and *k* is the rate at which the immune effector eliminates the pathogen *Aliivibrio*. *a*_*MA*_ and *a*_*AM*_ are the intraspecific competition coefficients. *s* and *m* are the toxin production and decay rates, and *e* is the rate at which toxin (or any other mechanism by the pathogen) inactivates the active immune effectors. *K*_*M*_(*I*), *I*_0_(*M*) are increasing saturating functions of *I* and *M*, in harmony with the assumptions that *Mycoplasma* activates the immune system to reach a higher equilibrium proliferation level, while in return the immune system of the host enhances the carrying capacity of the *Mycoplasma*:


$$\begin{array}{l} I_{0}\left(M\right)\;=\;I_{0}\left(1+\varepsilon \frac{M}{\sigma +M}\;\right),\\ K_{M}\left(I\right)=\;K_{M}\left(1+\delta \frac{I}{\beta +I}\;\right). \end{array}$$

Parameters ε and δ determine the maximal effect of *M* and *I* on *I*_0_ and *K*_*M*_, while β and σ are the half-saturation constants of these functions. If the host can only tolerate *Mycoplasma* (that is immune system neither support nor harm it), then δ and probably ε are zero in the previous functions. In this case, only the direct competition of *Mycoplasma* with *Aliivibrio* has to be taken into account, a situation that we will also analyze later.

We might assume that the dynamics of *I* and *T* are much faster than the dynamics of microbes (*s, m, r, e* ≫ *r*_*A*_, *r*_*M*_), that is, $\frac{dI}{dt}$= $0,\frac{dT}{dt}=0$ in Equations (1c,1d) (Rybicki et al., 2018). Consequently, the concentrations of immune effectors and toxins in steady-state are I^*^(*A, M*) = $\frac{I_{0}\left(1+\varepsilon \frac{M}{\sigma +M}\right)}{1+\frac{es}{mr}A},T^{*}$ = $\frac{s}{m}A.$

By substituting I^*^(A, M) and T^*^ into (1a, 1b), we receive the following dynamical system for *A* and *M*:


(2a) $$\begin{array}{r} \frac{dA}{dt}\;=\;r_{A}\left[\;1-A-a_{AM}M-I^{{}^{*}}\left(A,M\right)\right]A \end{array}$$

(2b) $$\begin{array}{r} \frac{dM}{dt}\;=\;r_{M}\left(1-\frac{M}{1+\delta \frac{I^{*}\left(A,M\right)}{\beta +I^{{}^{*}}\left(A,M\right)}}-a_{MA}A\right)M, \end{array}$$

where $I^{*}\left(A,M\right)\;=\;\frac{\pi \left(1+\varepsilon \frac{M}{\sigma +M}\right)}{1+\mu A},\;\pi \;=\;\frac{kI_{0}}{r_{A}},\;\mu \;=\;\frac{es}{rm},$ are variables, and other parameters are rescaled to $A\to AK_{A},\;M\to MK_{M},a_{AM}\to a_{AM}K_{M},\;a_{MA}\to a_{MA}K_{A},\sigma \to \frac{\sigma }{K_{M}}$. π is the relative immune efficiency, μ is the relative toxin efficiency, and *a*_*MA*_ and *a*_*AM*_ are the rescaled intraspecific competition coefficients, which are the key parameters of the model.

## Results

We examine the condition that the invading *Aliivibrio* could not spread if *Mycoplasma* is the dominant microbial resident of the host. According to the experimental observations, we assume that *Mycoplasma* arrives earlier in the distal intestine [typically in the early juvenile phase (Llewellyn et al., 2016)] than *Aliivibrio* and dominates in this section of the intestine before the infection. *Mycoplasma* reaches its equilibrium density, the stable fixed point of (2b) when *A* = 0. It is easy to show that 1 < M^*^ < 1+δ, the solution of $1-\frac{M}{1+\delta \frac{I*\left(0,,M\right)}{\beta +I^{*}\left(0,,M\right)}}\;=\;0$, is the only stable fixed point of (2b). *Aliivibrio* could not invade the *Mycoplasma* dominated microbiome if $\frac{dA}{dt}<0$ in the case of *A* ≈ 0 and *M* = M^*^. This leads to the following relation:


(3) $$\begin{array}{r} \left(1-a_{AM}M^{{}^{*}}-A\right)\left(1+\mu A\right)-\pi \left(1+\varepsilon \frac{M^{{}^{*}}}{\sigma +M^{{}^{*}}}\right)<0, \end{array}$$

which is satisfied if


(4) $$\pi >\pi_{0}(\alpha^{*},\varepsilon^{*})\;=\;\frac{1-\alpha^{*}}{1+\varepsilon^{*}},$$

where $\alpha^{*}\;=\;a_{AM}M^{*},\;\varepsilon^{*}\;=\;\varepsilon \frac{M^{*}}{\sigma +M^{*}}$. However, there are two different cases even if relation (4) is valid. If


(5) $$\begin{array}{r} \;\mu \;<\mu_{0}\left(\pi ,\alpha^{*},\varepsilon^{*}\right)\\ =\;\frac{2\pi \left(1+\varepsilon^{*}\right)-\left(1-\alpha^{*}\right)+2\sqrt{\pi \left(1+\varepsilon^{*}\right)\left(\pi \left(1+\varepsilon^{*}\right)-\left(1-\alpha^{*}\right)\right)}}{{\left(1-\alpha^{*}\right)}^{2}}, \end{array}$$

then *Aliivibrio* can never spread independently to its initial dose. However, if $\mu \geq \mu_{0}\left(\pi ,\alpha^{*},\varepsilon^{*}\right),$ then *Aliivibrio* spreads if its initial concentration is above a critical level. So, there is a critical dose of the pathogen above which it can infect the host. Naturally, if (4) is not valid, then $\frac{dA}{dt}>0$; thus, *Aliivibrio* always spreads independently to its initial concentration.

**Figure 3:**
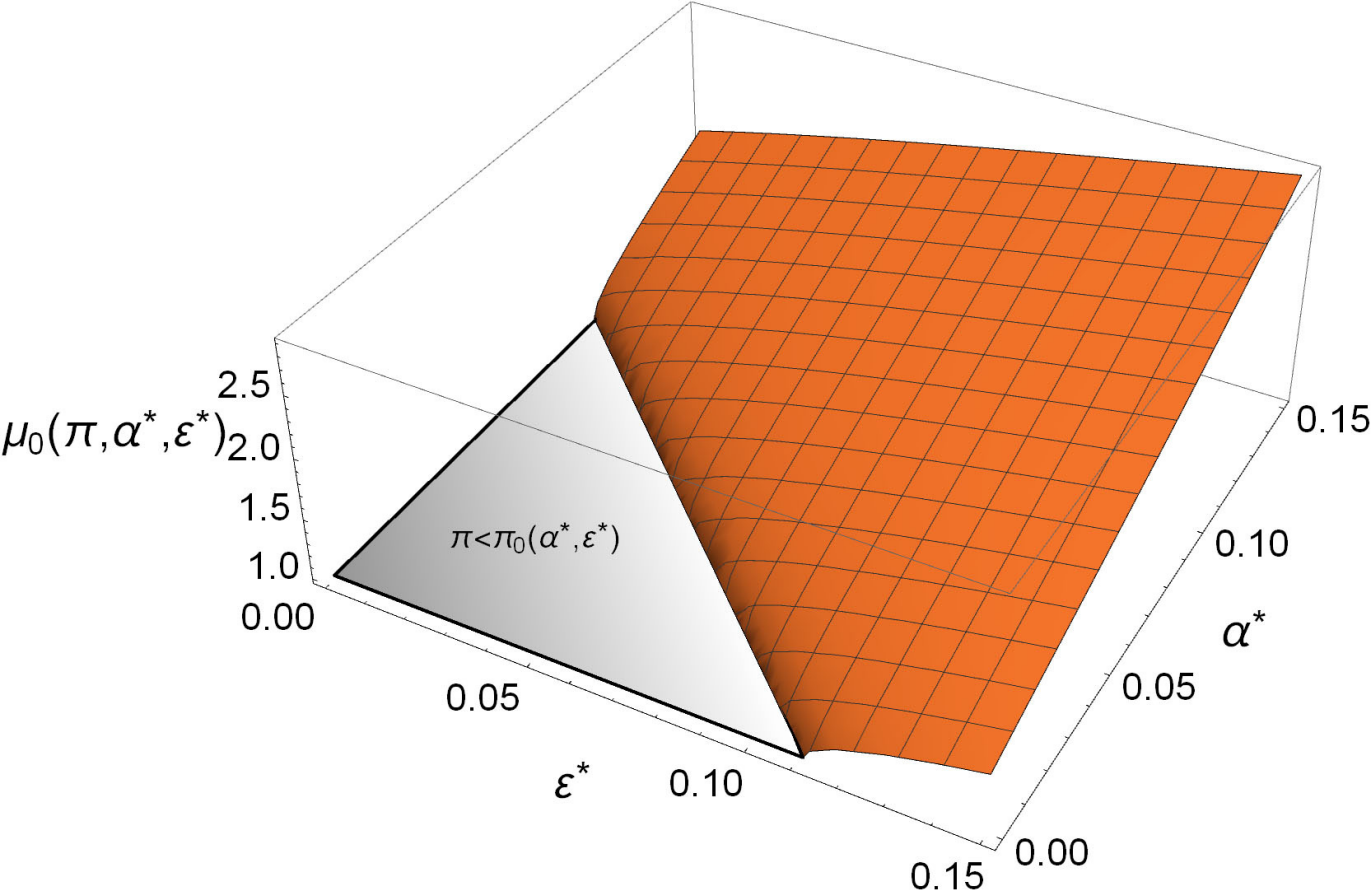
The critical toxin efficiency (μ_0_) in the function of direct competition (α^*^) and immune system facilitation (ε^*^). The gray triangle denotes the region where rare *Aliivibrio* always/spreads in the *Mycoplasma*-dominated microbiome [$\pi <\pi_{0}\left(\alpha^{*},\varepsilon^{*}\right)$]. The orange region covers the α^*^, ε^*^ values where rare *Aliivibrio* cannot invade. If the actual $\mu \;<\mu_{0}\left(\pi ,\alpha^{*},\varepsilon^{*}\right)$ which can be satisfied more easily when α^*^, ε^*^ are bigger, then the host is defended from the invasion of even a high dose of *Aliivibrio*, (π = 0.9).

*Mycoplasma* defends the host by having a direct competition with *Aliivibrio*, which is manifested in the parameter α^*^; however, it also benefits the host indirectly by facilitating its immune response, which is involved in the parameter ε^*^. Notably, the direct and the indirect effects both take a role in relations (4) and (5); that is, *Mycoplasma* not only prevents the rare *Aliivibrio* from spreading, but its presence increases the critical dose of *Aliivibrio* above which it can spread (see Figure 3).

According to the experimental results, Aliivibrio cannot expand in healthy hosts where *Mycoplasma* dominates the microbial abundance in the distal intestine (Bozzi et al., 2021) (Figure 1). Hence, this means that relation (4) and probably (5) are valid in most cases in healthy fishes. Assume that infection on the skin or any other external stress suppresses the immune system or decreases the health condition of the host, which causes a less effective immune reaction. Thus *I*_0_, and consequently π decreases. It leads to a decrease in M^*^, and thus in α^*^ and in ε^*^. Therefore, the right-hand side of (4) increases while the left-hand side decreases.

For the same reason, the right-hand side of (5) decreases too. Consequently, it can happen that π, the actual relative immune efficiency is no longer sufficiently high to prevent the spread of the pathogen. Alternatively, it can happen as well that the decrease of π and M^*^ keeps the relation (4) valid, but μ, the relative toxin efficiency becomes higher than the threshold level in (5), thus because of the stress a higher dose of pathogens can spread in the host.

The results presented by Bozzi et al. (2021) also suggest that *Mycoplasma* generally cannot spread if *Aliivibrio* becomes the dominant microbe in the distal intestine (Figure 1). This means in our model that $\frac{dM}{dt}<0$ if *M* ≈0 when *A* = A^*^ < 1 is at the equilibrium density. Substituting these values into (2b), we receive that $1-a_{MA}A^{*}<0$ guarantees that *Mycoplasma* invading in a low dose (rare invader) could not spread in an *Aliivibrio* dominated microbiota. Since A^*^ < 1, therefore *a*_*MA*_>1 is necessary to satisfy the previous relation. This means that the negative effect of *Aliivibrio* on *Mycoplasma* should be more intense than the negative effect of *Aliivibrio* on itself (since this constant is normalized to one in the model). This happens if the *Aliivibrio* species actively destroys the living conditions of *Mycoplasma*. Since most *Aliivibrio* strains produce toxin, it is conceivable that toxin harms *Mycoplasma* too, which mapped to the condition *a*_*MA*_>1 in our model. Contrary, if the competing efficiency of *Aliivibrio* is not strong enough, that is if $1-a_{MA}A^{*}>0$ then rare *Mycoplasma* can spread to the *Aliivibrio* dominated state.

**Figure 4:**
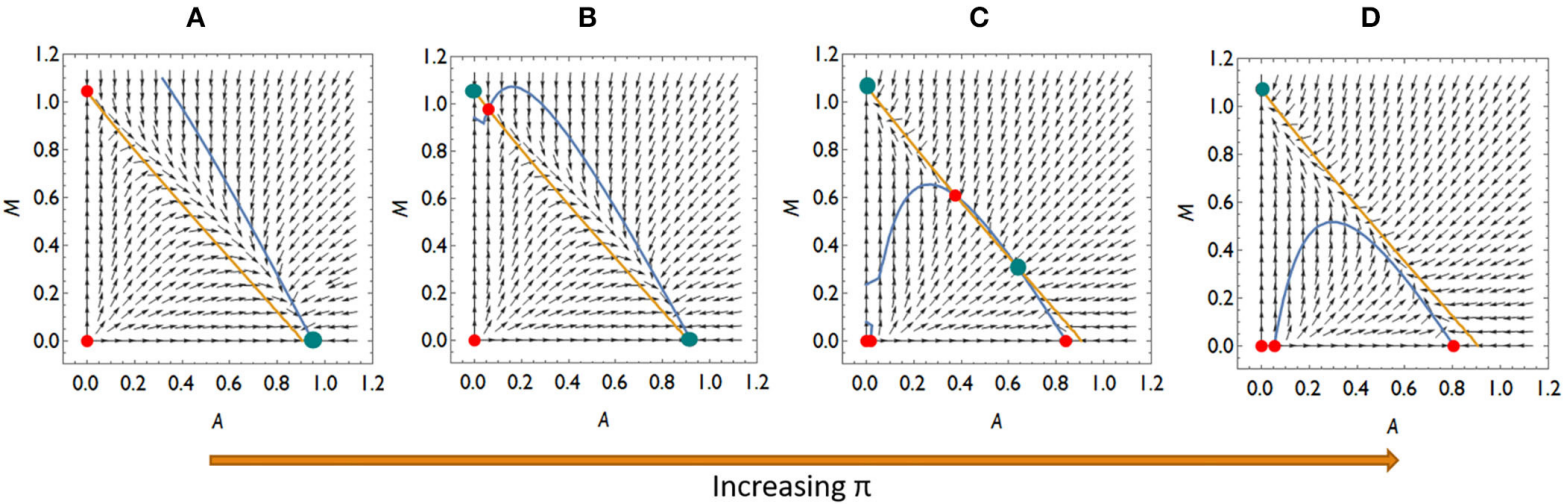
The qualitatively different dynamics of the *Mycoplasma–Aliivibrio* system when *Aliivibrio* deteriorate *Mycoplasma* living conditions. The nullclines of **(A,B)** are depicted (yellow dM/dt = 0, blue dA/dt = 0), so their intersections define the fixed points of the dynamics. Red points denote the unstable, while green points denote the stable fixed points of the system. At low relative immune efficiency (π) *Aliivibrio* dominates the dynamics **(A)**. At intermediate π the system is bistable **(B,C)**, while at high π *Mycoplasma* dominates the dynamics **(D)**. Parameters: (A) π = 0.4, (B) π = 0.6, (C) π = 1.1, (d) π = 1.3, other parameters are the same for all subfigures: *r*_*A*_ = *r*_*M*_ = 1, *a*_*AM*_ = 0.5 *a*_*MA*_ = 1.1, ε = 0.1, σ = 0.5, δ = 0.1, β = 0.5, μ = 7.

Collecting the possible invasion scenarios listed above, there are four qualitatively different competition situations if an invasion of *Mycoplasma* is not possible at lower π-s (higher A^*^) but possible at higher π-s (lower A^*^): (a) *Aliivibrio* is dominant over *Mycoplasma*, (b) neither rare invaders can spread, so the system is bistable, and (c) *Aliivibrio* can spread above a critical concentration while rare *Mycoplasma* can spread. The two species either are in stable coexistence or *Mycoplasma* is the winner of the competition, (d) *Mycoplasma* is dominant over *Aliivibrio* (Figure 4; Supplementary material).

Importantly, the microbial pattern experienced in Figure 1 can be explained by the behavior of our model. The stress decreases *I*_0_, thus it decreases π as well. This allows *Aliivibrio* to spread and dominate the total microbial abundance (Figure 4A). After the treatment, parameter *I*_0_ recovers leading to an increase of π again, but if this recovered π is not high enough, then *Aliivibrio* remains dominant (Figure 4B) or the two strains coexist after reinvasion of *Mycoplasma* (Figure 4C). The observation of different microbial states of the hosts after treatment (Figure 1) can be the consequence of different health states, such as the immune efficiency π, of the hosts which, as we have shown, can lead to different microbiome dynamics.

**Figure 5:**
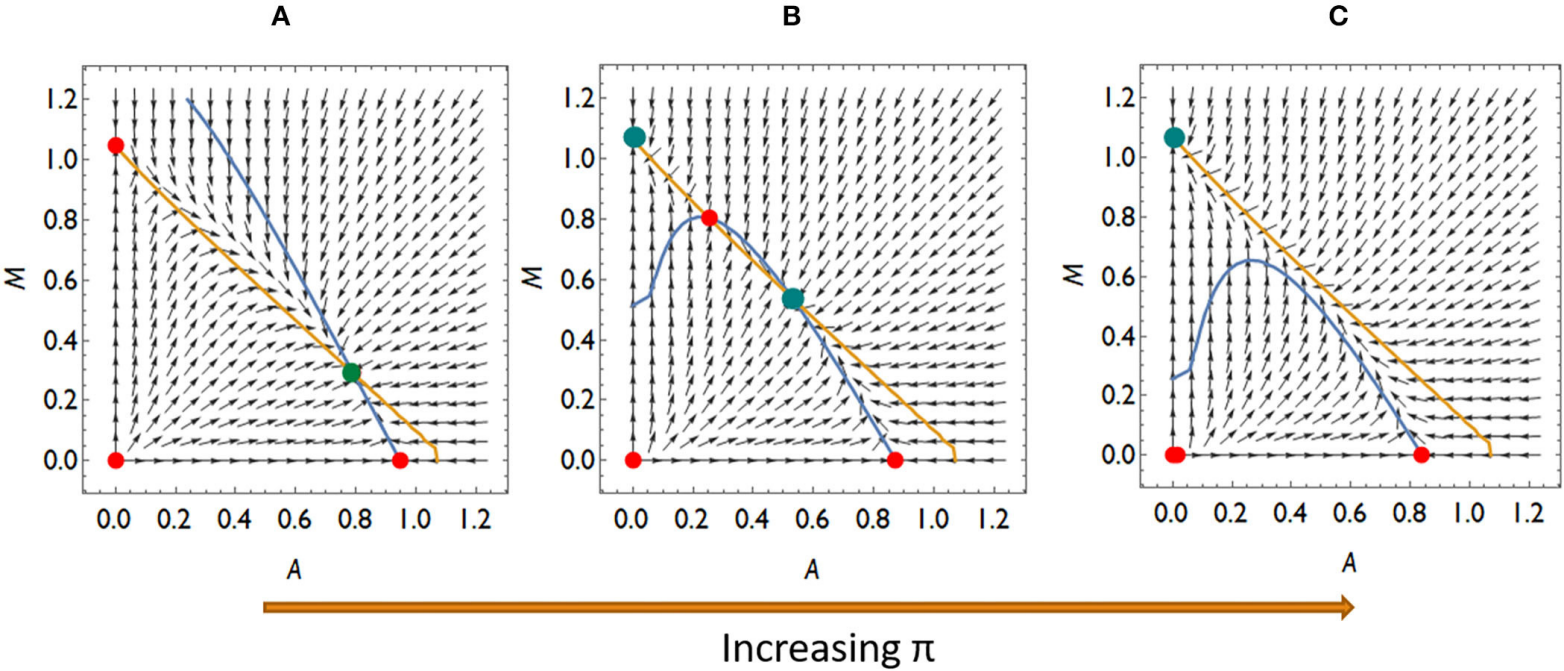
The qualitatively different dynamics of the *Mycoplasma–Aliivibrio* system when rare *Mycoplasma* always invades *Aliivibrio*. The nullclines of (1–2) are depicted (yellow dM/dt = 0, blue dA/dt = 0). Red dots denote the unstable fixed points, green ones denote the stable fixed points of the system. At low-relative immune efficiency (π), rare species invade and *Mycoplasma* are in coexistence with *Aliivibrio* **(A)**. At intermediate π the system is bistable **(B)**, while at high π *Mycoplasma* dominates the dynamics **(C)**. Parameters: (A) π = 0.4, (B) π = 0.9, (C) π = 1.1, (D) other parameters are the same for all subfigures: *r*_*A*_ = *r*_*M*_ = 1, *a*_*AM*_ = 0.5 *a*_*MA*_ = 0.9, ε = 0.1, σ = 0.5, δ = 0.1, β = 0.5, μ = 7 .

Let us also consider what dynamic cases are possible if *Aliivibrio* cannot significantly hamper the living conditions of *Mycoplasma* even though *Aliivibrio's* toxin suppresses the salmon host's immune system. Then *a*_*MA*_ < 1, thus rare *Mycoplasma* can always replace the resident *Aliivibrio* population. There are typically three different dynamical scenarios in this case. There is a stable coexistence of species for weak relative immune efficiency (Figure 5A), while the system is bistable with a coexistence or a *Mycoplasma* only stable state for intermediate relative immune efficiency (Figure 5B). For high relative immune efficiency, *Mycoplasma* will be dominant as in the previous scenario (compare Figure 4D with Figure 5C). Under these conditions, stress does not lead to the displacement of *Mycoplasma*. However, the coexistence of the strains is the expected outcome, which is still compatible with the experimental results for some individuals (see Figure 1). However, assuming this dynamic situation, the *Mycoplasma* concentrations should increase after the stress is removed (after treatment). But this is not what we see in the experiment. Therefore, we can assume that once *Aliivibrio* reaches a particular concentration, it negatively impacts the living conditions of *Mycoplasma*, a scenario following our analyses above (Figures 4A,B) as the typical case.

The model parameters determine which of the above scenarios will play out. The dynamical parameters can be considered constant in a given host–microbiome system, except π, the relative efficiency of the immune system, which can decrease and increase because of stress and treatment. As π increases, we can move from an *Aliivibrio*-dominated stable microbiome to a *Mycoplasma* dominated state *via* bistable behavior, including the stable coexistence of *Mycoplasma* and *Aliivibrio*.

Since the coexistence of *Mycoplasma* and *Aliivibrio* after the stress is occasionally observed and the reinvasion of *Mycoplasma* after the treatment is experienced too, although it is not typical in the Atlantic salmon experiment (Figure 1), it is likely that *Aliivibrio* actively harms *Mycoplasma* (*a*_*MA*_>1). However, according to the experimental results depicted in Figure 1, stress decreases π to a value where *Aliivibrio* becomes dominant (Figure 4A), and after treatment, it can increase only to allow a bistable state (Figures 5B,C) in most cases. Naturally, these observations do not exclude that the scenario presented in Figure 5 occurs in other salmonid-related *Mycoplasma* pathogen systems.

### *Mycoplasma* does not facilitate the immune system, and the immune system does not increase *Mycoplasma* concentration

We consider here the case when *Mycoplasma* and the host conform to a mutualistic interaction or where the host tolerates *Mycoplasma*. However, the immune system is not facilitated by *Mycoplasma*, that is, ε = 0 in the model. These modifications did not lead to qualitative changes compared to the previous analysis. The difference is only quantitative, making *Mycoplasma* stable against invasion of *Aliivibrio* in a narrower parameter space [see Equations (4 and 5)]. Similarly, suppose the host immune system does not increase the carrying capacity of *Mycoplasma* directly; that is, when δ = 0, then M^*^ = 1, which again does not change the previous derivations except that the invasion of *Aliivibrio* will be more likely [see Equations (4 and 5)].

### The invader species (*Aliivibrio*) does not suppress the host immune system

To make a comprehensive analysis, we consider the situation when *Aliivibrio* does not harm the efficiency of the immune system directly. This means formally in the model that μ = 0. The consequence is that the dosage effect disappears in the system; that is, rare *Aliivibrio* simply cannot invade the resident *Mycoplasma* if Equation (4) is valid, and invades if this relationship does not hold. Since it is assumed that *Aliivibrio* does not produce a toxin, *Aliivibrio* does not deteriorate *Mycoplasma* habitat, that is *a*_*MA*_ < 1 (intraspecific competition is more robust than interspecific) should be valid in the model. So, *Mycoplasma* invariably invades the *Aliivibrio*-dominated community (*dM*/*dt*>0 if *M* ≈0 and *A* = A^*^ < 1).

Two different dynamical outcomes are possible, either *Mycoplasma* dominates for stronger relative immune efficiency [π is bigger, Equation (4) is invalid], or the two competing strains are in coexistence [π is lower, Equation (4) is valid]. Consequently, stress never leads to *Aliivibrio* dominance which contradicts the experimental results presented in Figure 1.

## Discussion

We present one of the first models able to describe a, albeit simple, complete intestinal microbiome community of a vertebrate host. Our model stands out from predecessors by considering realistic parameters of the host immune function, a mutualist microbe able to induce host immune reactions, and a toxin-producing pathogenic microbe. The dynamics explained by our model are in line with multiple empirical observations (Table 1).

Based on the experimental observations described, we assumed that salmon and *Mycoplasma* form a mutualistic relationship in a way that the immune system of the host increases the carrying capacity of *Mycoplasma* in the distal gut, and vice versa, the presence of *Mycoplasma* can boost the immune response of the host. Furthermore, we assume that *Aliivibrio* represents any toxin-producing intestinal pathogen of salmonids. *Mycoplasma* is believed to colonize the intestine of salmon in the juvenile phase before the *Aliivibrio* can infect it. *Mycoplasma* and *Aliivibrio* compete in the distal intestine, where *Aliivibrio* can be toxic for Mycoplasma, which is also considered in the model. The last assumption of the model is that infection or other stress factors elicit an acute immune response that will remove resources from other metabolic processes in the host fish.

Analyzing the mathematical model of the above system, we have shown that *Mycoplasma* helps to prevent the host from the *Aliivibrio* infection. If relative immune efficiency is high enough, *Aliivibrio* cannot invade (Figures 4D, 5C). Suppose the host is infected or stressed in any way that leads to an immuno-deprived state, or the *Mycoplasma* density reduces for any reason, then *Aliivibrio* can spread and replace *Mycoplasma* (Figures 4A,B). We have shown that if *Aliivibrio* becomes dominant in the distal intestine, then *Mycoplasma* cannot invade in low concentrations if the toxin harms *Mycoplasma* growth (Figure 4B). The system is bistable in a wide range of relative immune efficiency: depending on the parameters, the two stable states are: *Mycoplasma* only and *Aliivibrio* only (Figure 4B) or *Mycoplasma* only and coexistence of *Mycoplasma* and *Aliivibrio* (Figures 4C, 5B). The system flips from the *Mycoplasma* only state to the other one if the invader *Aliivibrio* concentration is high enough. *Mycoplasma* and the host immune system define that critical level of invasion. Together, this prevents the pathogen from spreading easily in a way that, besides the level of relative immune efficiency, the level of mutualism helps the competitive ability of *Mycoplasma* involved in the protection from the pathogen (Figure 3). We emphasize here that the behavior of the model explains the observations of a previous experiment (Figure 1). Furthermore, while *Mycoplasma–Aliivibrio* dominant microbiomes are widespread in salmonid hosts (Table 1), it is highly likely that the dynamics covered by our model are common in both these economically important and numerous related species. Our analysis points out that, due to the bistability of the system, the *Aliivibrio* dominant state can only be eliminated by introducing high doses of *Mycoplasma*. A possible solution would be to feed the individuals infected by *Aliivibrio* with the gut content (or shredded intestine) of healthy individuals carrying high intestinal biomass of *Mycoplasma* sp.

Since some assumptions of the model are based only on indirect observations, consequently we examined the robustness of the model to these assumptions. We have shown that the dynamical behavior does not change qualitatively if the immune system of the host and *Mycoplasma* do not help each other directly (ε = 0, δ = 0); however, the presence of mutual help (ε>0, δ>0) increases the range of conditions where the *Mycoplasma* dominated state is stable against invasion of *Aliivibrio*. Similarly, the dosage effect, the possibility of mutual invasion of *Mycoplasma* and *Aliivibrio*, and stable coexistence of them are possible even if *Aliivibrio* does not harm *Mycoplasma* effectively (Figures 4A,B). Contrary, in a model where *Aliivibrio* does not harm the immune system, the Allee effect (invasion only above a critical concentration of *Aliivibrio*) disappears. Naturally, outer stress suppressing the immune system still facilitates invasion of the pathogen, but successful invasion always leads to the coexistence of *Mycoplasma* and *Aliivibrio*, which is not compatible with the results of (Bozzi et al., 2021) (see Figure 1).

Naturally, there are simplifications of the study. First, it should be stated that the *Mycoplasma* component in our model represents a single dominant species following the observations listed in Table 1, whereas numerous distinct species of *Mycoplasma* may be associated with their fish hosts, including the skin tissue (Cheaib et al., 2021b). The model neglects the spatial constraints and heterogeneities present in the gut and the non-even distributions of the cells and materials by the finite speed of diffusion of these materials and cells. Based on previous studies, however, it is highly probable that we do not lose the essence of the dynamics with these simplifications (compare e.g., Scheuring and Yu, 2012) with (Boza et al., 2019). To make the model tractable, we consider only the dominant species of the community, and the immune system dynamics are highly simplified. While this is an excellent first step toward developing models that help us move from only studying host–microbe and microbe–microbe interactions to better understand host–microbe–microbe interactions, the effect of our simplifications needs to be further explored in the context of species with more complex gut microbiome communities.

In summary, our model robustly describes the patterns seen in the experiments and remains consistent with other experimental observations. Based on the model, it is expected that the unfavorable *Aliivibrio* dominated microbiome community after stress can, in most cases, only be restored to a favorable *Mycoplasma* dominated state by introducing a high dose of *Mycoplasma*. We propose to test this specific hypothesis and the broader relevance of our model in future experiments.

## Data availability statement

The original contributions presented in the study are included in the article/Supplementary material, further inquiries can be directed to the corresponding author/s.

## Author contributions

IS designed and analyzed the mathematical model. IS, JR, DB, and ML wrote the paper. IS and ML designed the project. DB crafted Figure 1. DB and JR performed the literature research for Table 1. All authors contributed to the article and approved the submitted version.

## Funding

The research was funded by the Hungarian National Fund, NKFI (K128289) to IS, the Independent Research Fund Denmark (HappyFish, Grant No. 8022-00005B), the Danish National Research Foundation Grant No. DNRF143 to ML, and the Two European Union's Horizon 2020 Actions HoloFood (817729) to ML as well as FindingPheno (952914) to IS and ML.

## Conflict of interest

The authors declare that the research was conducted in the absence of any commercial or financial relationships that could be construed as a potential conflict of interest.

## Publisher's note

All claims expressed in this article are solely those of the authors and do not necessarily represent those of their affiliated organizations, or those of the publisher, the editors and the reviewers. Any product that may be evaluated in this article, or claim that may be made by its manufacturer, is not guaranteed or endorsed by the publisher.
