# Supplementary material for: A strategic model of a host–microbe–microbe system reveals the importance of a joint host–microbe immune response to combat stress-induced gut dysbiosis
Source: Front Microbiol. 2022 Aug 4;13:912806. doi: 10.3389/fmicb.2022.912806 (PMC9386248; doi:10.3389/fmicb.2022.912806)
Supplement: Supplementary file 1 [file Data_Sheet_1.docx]

**Supplement**

Here we show that null clines of (2a) and (2b) intersect each other in at most three points determining one stable and two unstable inner fixed points. However, in order for the curves to intersect at three points, fine-tuning and very specific parameters are needed. Without fine-tuning, the zero clines intersect in at most two points, so there are a maximum of one unstable and one stable inner fixed point of the dynamics as it is demonstrated in Figure 3.

The $(A^{*}>0,M^{*}>0),$ fixed points are the solutions of

$F_{1}(A,M)= 1-A-a_{AM}M-I^{*}(A,M)=0$ (A1)

$F_{2}(A,M)=1-\frac{M}{1+\delta\frac{I*(A,M)}{\beta+I^{*}(A,M)}}-a_{MA}A=0$, (A2)

which points are the intersections of the $F_{1}(A,M)=0, F_{2}(A,M)=0$ curves in the $A>0, M>0$ sub-plane. To determine the number of possible intersections of these curves we analyze them qualitatively.

Using the definition of total derivative, and since $dF_{i}(A, M)=0$, we can compute the derivative of *M* according to *A* in both equations as

$\frac{dM}{dA}=\frac{-\frac{\partial F_{i}}{\partial A}}{\frac{\partial F_{i}}{\partial M}}$ , where *i*=1,2. (A3)

Substituting (A2) into (A3) we receive that

$\frac{dM}{dA}=\frac{a_{MA}(1+\delta J^{*}(A,M))^{2}-\delta M\frac{\partial J^{*}(A,M)}{\partial A}}{-1-\delta J^{*}(A,M)+\delta M\frac{\partial J^{*}(A,M)}{\partial M}} ,$ (A4)

where $J^{*}(A,M)=\frac{I*(A,M)}{\beta+I^{*}(A,M)}.$ Since ${1+{\delta J}^{*}(A,M)}>0$and $\partial J^{*}/\partial A<0,$ the nominator of the right hand side of (A4) is positive. By explaining the denominator it will be

$-1 -\delta\frac{1}{\beta+I} I^{*} +\delta\frac{1}{\beta+I^{*}}\left[ \frac{\beta}{\beta+I^{*}}\frac{\sigma}{\sigma+M}(I^{*}-\frac{\pi}{1+\mu A}) \right]$ , which is negative, since the third term is always smaller than the second term. Consequently, the derivative is always negative, so the $F_{2}(A,M)=0$ determines an always decreasing $M(A)$ curve in the $(A,M)$ plane. Further, it can be shown that the curve is convex. For that we have to study the sign of the second derivative of $M(A)$ which is

$\frac{d^{2}M}{dA^{2}}=\frac{\partial}{\partial A}\left( \frac{dM}{dA} \right)+\frac{dM}{dA}\frac{\partial}{\partial M}\left( \frac{dM}{dA} \right).$ (A5)

Substituting (A4) into (A5) and performing the operations we receive that

$\frac{d^{2}M}{dA^{2}}=\frac{-(a_{MA}(1+\delta J^{*}(A,M))^{2}-\delta M\frac{\partial J^{*}(A,M)}{\partial A})(-\delta\frac{\partial J^{*}(A,M)}{\partial A}+\delta M\frac{\partial{J^{*}}^{2}(A,M)}{\partial M\partial A})}{(-1-\delta J^{*}(A,M)+\delta M\frac{\partial J^{*}(A,M)}{\partial M})^{2}}+\frac{{2a}_{MA}\delta(1+\delta J^{*}(A,M)\frac{\partial J^{*}(A,M)}{\partial A}-\delta M\frac{\partial{J^{*}}^{2}(A,M)}{\partial A^{2}}}{-1-\delta J^{*}(A,M)+\delta M\frac{\partial J^{*}(A,M)}{\partial M}}+$

$-\left( \frac{a_{MA}(1+\delta J^{*}(A,M))^{2}\delta M\frac{\partial{J^{*}}^{2}(A,M)}{\partial M^{2}}-\delta M\frac{\partial J^{*}(A,M)}{\partial A}}{(-1-\delta J^{*}(A,M)+\delta M\frac{\partial J^{*}(A,M)}{\partial M})^{2}}+\frac{{2a}_{MA}\delta(1+\delta J^{*}(A,M)\frac{\partial J^{*}(A,M)}{\partial M}-\delta M\frac{\partial{J^{*}}^{2}(A,M)}{\partial A\partial M}-\delta\frac{\partial J^{*}(A,M)}{\partial A}}{-1-\delta J^{*}(A,M)+\delta M\frac{\partial J^{*}(A,M)}{\partial M}} \right)\times$ $\frac{a_{MA}(1+\delta J^{*}(A,M))^{2}-\delta M\frac{\partial J^{*}(A,M)}{\partial A}}{-1-\delta J^{*}(A,M)+\delta M\frac{\partial J^{*}(A,M)}{\partial M}}$ . (A6)

Knowing that $a_{MA}$ and $\delta$ are positive and $\partial J^{*}/\partial A<0,$ $\partial J^{*}/\partial M>0$, $\partial{J^{*}}^{2}/\partial M^{2}<0$ $\partial^{2}J^{*}/\partial A^{2}>0,\partial^{2}J^{*}/\partial{A\partial M}<0$, it can be shown after tiresome but simple calculations that $\frac{d^{2}M}{dA^{2}}>0,$ thus the $M(A)$ curve of $F_{2}(A,M)=0$ is convex.

Now we focus on the $F_{1}(A,M)=0$ equation. By substituting (A1) into (A3), and performing the derivation we receive that

$\frac{dM}{dA}=-\frac{1+\frac{\partial I^{*}}{\partial A}}{a_{AM}+\frac{\partial I^{*}}{\partial M}}$.

It is easy to show that $\partial I^{*}/\partial M>0$ for $0 \leq M$, consequently the denominator is always positive, and the sign of the derivative is determined by the sign of the nominator, that is $sign(dM/dA)=-sign(1+\partial I^{*}/\partial A)$. The monotonicity of the function *I** ensures that for each value *A* there is a single value *M* satisfying the $F_{1}(A,M)=0$ relation. We can compute the $sign(dM/dA)$ for every (*A, M*) pair satisfying $F_{1}(A,M)=0$. Let us consider the $F_{1}(0,\hat{M})=0$ point on the curve. Assume that $1<$ $-\partial I^{*}/\partial A|(0,\hat{M)}=\pi\mu(1+\varepsilon\hat{M}/(\sigma+\hat{M}))$. Thus $dM/dA>0$ at this point, that is $M(A)$ increases locally. Let us consider now the $F_{1}(\hat{A},0)=0$ point of the curve. Here $\partial I^{*}/\partial A|(\hat{A,}0)=0,$ thus $dM/dA<0 , M(A)$ always decreases locally at this point. Since $\partial I^{*}/\partial A<0$ if $M>0$ thus there is only one $(A_{max},M_{max}$) point where $dM/dA=$ $1+\partial I^{*}/\partial A|(A_{max},M_{max})=0$, and this is the only maximum point of the $M(A)$ curve of $F_{1}(A,M)=0$. Naturally if $1>$ $-\partial I^{*}/\partial A|(0,\hat{M)}$ then $dM/dA<0$for every $A\geq0, M\geq0$ points satisfying the $F_{1}(A,M)=0$ equation.

Since $F_{1}(A,M)=0$ defines a curve with one maximum point and $F_{2}(A,M)=0$ determines an $M(A)$ curve which is always decreasing in the (A, M) coordinate system, therefore, if the two curves intersect at more than one point, there must be at least one intersection at the point on the section of the curve $F_{1}(A,M)=0$ where *M* decreases in function of *A*, that is where $1+\frac{\partial I^{*}}{\partial A}>0$ (Fig. S1).


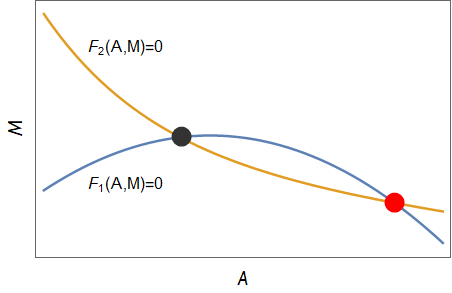


Figure S1. Schematic figure of intersections of $F_{1}(A,M)=0, F_{2}(A,M)=0$ curves on the $(A, M)$coordinate system. Since $F_{1}$has only one maximal point and $F_{2}$ is a strictly monotonously decreasing (convex) function, one of the intersections of the curves should be in the region where $F_{1}$ is decreasing (red point).

To determine the concavity-convexity of $F_{1}(A,M)=0$ we have to study the sign of the second derivative of $M(A)$ as previously. Substituting $dM/dA$, performing the derivations and making some simplifications we receive that

$\frac{d^{2}M}{dA^{2}}=\frac{-1}{\left( a_{MA}+\frac{\partial I^{*}}{\partial M} \right)^{3}}\left( \frac{\partial^{2}I^{*}}{\partial M^{2}}\left( 1+\frac{\partial I^{*}}{\partial A} \right)^{2}+a_{MA}+\frac{\partial I^{*}}{\partial M} \right)\left( \left( a_{MA}+\frac{\partial I^{*}}{\partial M} \right)\frac{\partial^{2}I^{*}}{\partial A^{2}}-2\left( 1+\frac{\partial I^{*}}{\partial A} \right)\frac{\partial^{2}I^{*}}{\partial{M\partial A}} \right)$ .

Knowing that $a_{MA}$ is positive and $\partial I^{*}/\partial A<0,$ $\partial I^{*}/\partial M>0$,$\partial^{2}I^{*}/\partial A^{2}>0,$ $\partial^{2}I^{*}/\partial M^{2}<0, \partial^{2}I^{*}/\partial{A\partial M}<0$then $\frac{d^{2}M}{dA^{2}}<0$, and consequently $F_{1}(A,M)=0$ is concave to the right of the maximal point of the curve if

$\frac{\partial^{2}I^{*}}{\partial M^{2}}\left( 1+\frac{\partial I^{*}}{\partial A} \right)^{2}+a_{MA}+\frac{\partial I^{*}}{\partial M}>0.$

Explaining the derivatives and rearranging the inequality the above condition leads to

$\frac{\frac{\left( \sigma+M \right)^{3}}{\sigma}\frac{a_{MA}}{\pi\varepsilon}\left( 1+\mu A \right)+\sigma+M}{\left( 1-\pi\left( 1+\varepsilon\frac{M}{\sigma+M} \right)\frac{\mu}{\left( 1+\mu A \right)^{2}} \right)^{2}}>1.$ (A7)

Since $\pi, \mu, a_{MA}$are around one, and the perturbation parameters $\sigma, \varepsilon$ are typically much smaller than one, the previous relation is generally valid. This means that the convex $F_{2}(A,M)=0$ curve and the concave $F_{1}(A,M)=0$ curve can intersect each other in at most two points (see e.g. Fig 3c and Fig S1). The concavity of $F_{1}(A,M)=0$ to the right of the $(A_{max},M_{max})$ point can be invalid only if $\sigma\ll\varepsilon$, and $M\approx0$ in (A7). Then the concave curve can become convex which allows the presence of an additional intersection between $F_{1}(A,M)=0, F_{2}(A,M)=0$ curves. In total, there can be two unstable and one stable inner fixed points of the dynamics. However, since the new unstable fixed point emerges at $M\approx0$, it practically cannot prevent the spread of rare *M* (Fig. S2). It is also true that the case presented in Fig. S2 only occurs for very specific fine tuning of the parameters, so it can be excluded from the general analysis.


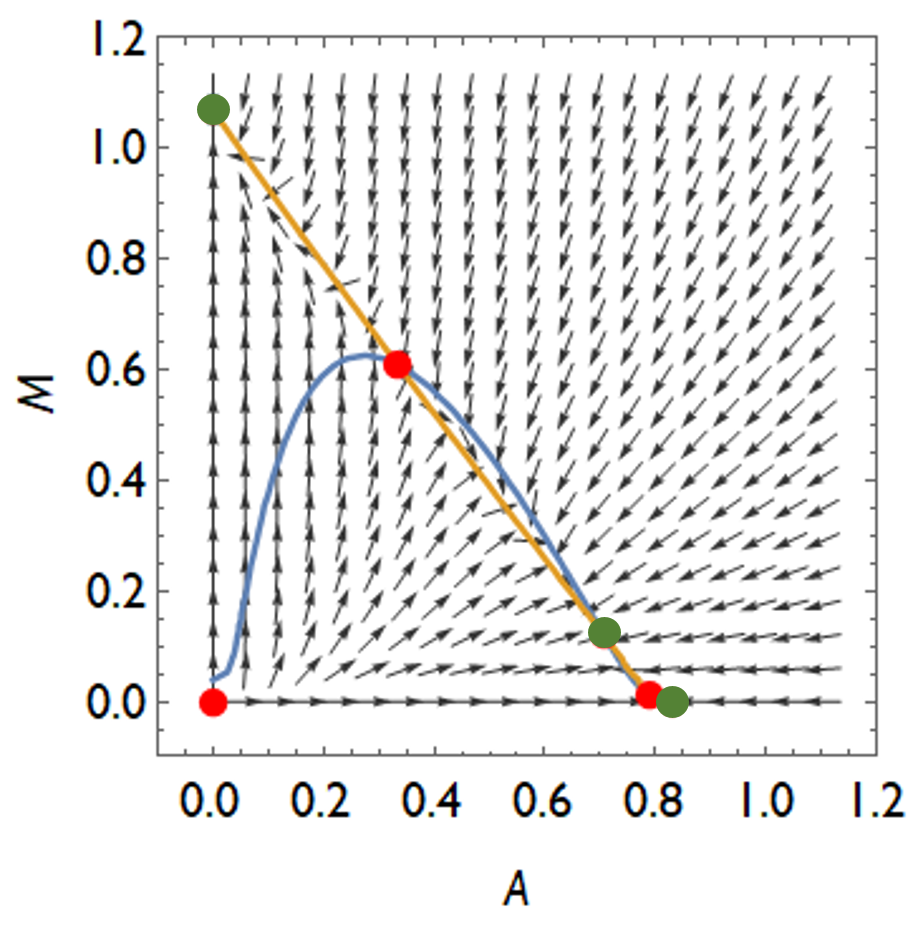


Figure S2. *Mycoplasma-Aliivibrio* dynamics when the system is multistable. There are two unstable inner fixed points beside the stable inner fixed point. The nullclines of (1-2) are depicted (yellow *dM/dt*=0, blue *dA/dt*=0). Red dots denote the unstable fixed points, green ones denote the stable fixed points of the system. $r_{A}=r_{M}=1, a_{AM}=0.4, a_{MA}=1.25$, $\varepsilon=0.3, \sigma=0.01, \delta=0.1 ,\beta=0.5 , \mu=5,$ $\pi=0.87.$
